# Sex-Specific Differences of the Inflammatory State in Experimental Autoimmune Myocarditis

**DOI:** 10.3389/fimmu.2021.686384

**Published:** 2021-05-28

**Authors:** Maria Luisa Barcena, Sarah Jeuthe, Maximilian H. Niehues, Sofya Pozdniakova, Natalie Haritonow, Anja A. Kühl, Daniel R. Messroghli, Vera Regitz-Zagrosek

**Affiliations:** ^1^Department of Geriatrics and Medical Gerontology, Charité – Universitätsmedizin Berlin, Corporate Member of Freie Universität Berlin, Humboldt-Universität zu Berlin and Berlin Institute of Health, Berlin, Germany; ^2^DZHK (German Centre for Cardiovascular Research), Berlin Partner Site, Berlin, Germany; ^3^Department of Internal Medicine – Cardiology, Deutsches Herzzentrum Berlin, Berlin, Germany; ^4^Climate and Health Program (CLIMA), Barcelona Institute for Global Health (ISGlobal), Barcelona, Spain; ^5^iPATH Berlin-Immunopathology for Experimental Models, Charité – Universitätsmedizin Berlin, Corporate Member of Freie Universität Berlin, Humboldt – Universität zu Berlin and Berlin Institute of Health, Berlin, Germany; ^6^Department of Internal Medicine and Cardiology, Charité - Universitätsmedizin Berlin, Berlin, Germany; ^7^Institute for Gender in Medicine, Center for Cardiovascular Research, Charité - Universitätsmedizin Berlin, Corporate Member of Freie Universität Berlin, Humboldt - Universität zu Berlin and Berlin Institute of Health, Berlin, Germany; ^8^Department of Cardiology, University Hospital Zürich, University of Zürich, Zürich, Switzerland

**Keywords:** sex differences, inflammation, experimental autoimmune myocarditis, cytokines, cardiac dysfunction

## Abstract

Increasing evidence suggests male sex as a potential risk factor for a higher incidence of cardiac fibrosis, stronger cardiac inflammation, and dilated cardiomyopathy (DCM) in human myocarditis. Chronic activation of the immune response in myocarditis may trigger autoimmunity. The experimental autoimmune myocarditis (EAM) model has been well established for the study of autoimmune myocarditis, however the role of sex in this pathology has not been fully explored. In this study, we investigated sex differences in the inflammatory response in the EAM model. We analyzed the cardiac function, as well as the inflammatory stage and fibrosis formation in the heart of EAM male and female rats. 21 days after induction of EAM, male EAM rats showed a decreased ejection fraction, stroke volume and cardiac output, while females did not. A significantly elevated number of infiltrates was detected in myocardium in both sexes, indicating the activation of macrophages following EAM induction. The level of anti-inflammatory macrophages (CD68+ ArgI+) was only significantly increased in female hearts. The expression of Col3A1 and fibrosis formation were more prominent in males. Furthermore, prominent pro-inflammatory factors were increased only in male rats. These findings indicate sex-specific alterations in the inflammatory stage of EAM, with a pro-inflammatory phenotype appearing in males and an anti-inflammatory phenotype in females, which both significantly affect cardiac function in autoimmune myocarditis.

## Introduction

Myocarditis is a cardiovascular disease that is associated with myocardial inflammation and infiltration of immune cells into the heart muscle (1). Of those immune cells, it is predominantly macrophages and T-cells that infiltrate the cardiac tissue during viral or toxic injury in myocarditis (1–3). Impaired regulation of the autoimmune response against auto-myocardial proteins can lead to chronic inflammation followed by fibrosis, dilated cardiomyopathy (DCM), and heart failure at the end stage of myocarditis (4, 5). Mice infected with coxsackievirus B3 (CVB3) develop a chronic myocarditis, associated with the presence of anti-myosin autoantibodies, myocardial fibrosis, and cardiac remodeling (6–8), leading to alterations in the extracellular matrix (ECM) (9). In addition, mice or rats immunized with cardiac myosin and Complete Freund’s Adjuvant (CFA) exhibit experimental autoimmune myocarditis (EAM) (10, 11). Pro-inflammatory cytokines e.g., interleukin (IL)-6, IL-1β and tumor necrosis factor α (TNF-α) together with enhanced reactive oxygen species (ROS) production play a crucial role in the development of autoimmune myocarditis (7, 12). In the EAM model, male animals show an increased fibrotic remodeling of cardiac tissue, which is linked to DCM development (13). Moreover, male animals develop cardiac autoimmunity and chronic inflammation more often than females (14).

Sex differences in cardiovascular diseases leading to heart failure have been well documented (15, 16). Interestingly, men show higher prevalence and severity of cardiovascular diseases than premenopausal women (17–19). However, the risk of negative cardiovascular events increases in women after menopause (20). The male sex is more susceptible to the development of DCM or heart failure due to impaired cardiac remodeling and the cardiac response to stress (21, 22). Furthermore, female mice show less acute inflammation compared to male mice in a viral myocarditis model, although the rate of viral replication is not significantly different between the sexes (23, 24). Several pathological conditions in the heart are associated with increased testosterone levels, promoting increased collagen deposition, fibrosis formation, and remodeling of the ECM (25–28). Fibroblasts are responsible for preserving ECM balance (29–31). In cardiac tissue, the most prominent collagen fibers are collagen type I and collagen type III (32). Sex-related differences, regulated by sex hormones such as estrogen and testosterone, are also observed in the immune system (33, 34). In turn, the immune system also regulates sex hormone production and secretion (33). Sex hormones have an effect on cardiomyocytes, endothelial cells and fibroblasts and dramatically modulate the tissue response to inflammation in a sex-dependent manner (35, 36), e.g., *via* p38 and ERK signaling (37). It is interesting to note that male animals have a higher number of classically activated M1 macrophages, whereas females develop a population of alternatively activated TIM3-positive M2 macrophages (38, 39). Moreover, male mice can present a M2 macrophage subpopulation, which expresses the M1 macrophage marker toll-like receptor (TLR4) and IL-1β. It has been proposed that this M2 macrophage population is strongly involved in fibrotic remodeling of cardiac tissue (6, 40). Furthermore, estrogen decreases TNF-α expression in peripheral blood mononuclear cells (PBMC) (33, 41, 42) and increased TNF-α secretion was detected in premenopausal women who underwent oophorectomy (43). In contrast, testosterone induces a TH1-type immune response in both humans and rodents (23, 24, 44–46). Macrophages activate fibroblasts *via* TGF-β, platelet-derived growth factor (PDGF), and TNF-α (47, 48). Activated fibroblasts produce ECM and favor fibrosis formation after cardiac damage (49, 50). Even though these sex differences in molecular and cellular mechanisms in the immune system are well documented, their interplay in specific diseases is not yet fully understood.

In this study, we investigated sex-related alterations in the inflammatory state in EAM accompanied by fibrosis formation and decreased cardiac function. The functional analyses revealed an impaired cardiac function in male but not female animals. Sex differences were also found in macrophage polarization and fibrosis formation. EAM is associated with an increased expression of inflammatory markers in male hearts.

## Material and Methods

### Animals

Lewis rats were housed in cages with controlled temperature and humidity on a 12h light/12h dark cycle. They were kept in groups of four or five with free access to food and water. Male and female rats (age: male: 42-56 days and female: 50-80 days; body weight 230-260g, n=16) (Janvier, Le Genest-St-Isle, France) were immunized as previously described with a myosin dose of 0.25 mg to the rear food pads on day 0 (51). 21 days later the animals were euthanized; their hearts, spleens, tibias, lungs, livers, and kidneys were extracted and snap frozen in liquid nitrogen and stored at -80°C. Non-immunized Lewis male and female rats were used as the control (n=10). All procedures and experimental protocols were performed in accordance with the Guide for the Care and Use of Laboratory Animals published by the U.S. National Institutes of Health and were approved by the relevant local authorities (Landesamt für Gesundheit und Soziales).

### Cardiac Magnetic Resonance Imaging

Cardiac function was evaluated by electrocardiographically triggered cardiac magnetic resonance imaging (CMR) as described in an earlier study (13). Left ventricular ejection fraction, end-diastolic volume, end-systolic volume, and cardiac output were measured before, 14 days, and 21 days after immunization.

### Analysis of Heart Weight to Body Weight Ratio

Body weight (BW) was measured before performing CMR. After euthanasia, the hearts without atria were weighed, and the relative heart weight (HW) to body weight (BW) ratio (HW/BW) was calculated as described in (13).

### Analysis of Muscle Hypertrophy and Immune Cell Infiltrate in Heart Tissue

Using the H&E staining, heart muscle hypertrophy score and the amount of immune cell infiltrates was counted in myocardium from male and female immunized and non-immunized rats (n=12).

### Immunohistochemistry

Paraffin-embedded cardiac tissue sections were incubated with anti-arginase 1 (clone N-20, 1:100, Santa Cruz, USA) primary antibody followed by incubation with secondary antibody biotinylated rabbit anti-goat (1: 400, Dianova, Germany). Biotin was detected with alkaline phosphatase-labelled streptavidin (Agilent, USA) and visualized using RED (Agilent, USA) as a chromogen. Proteins and enzymes were inactivated with heat and alkaline pH prior to incubation with anti-CD68 (1:250, Amsbio #1518), followed by incubation with Alexa488-labelled secondary antibody (1:400, donkey anti-rabbit, Invitrogen, Germany). DAPI (Sigma, Germany) was used to stain nuclei and sections mounted with Fluoromount-G (Southern Biotech, USA). Negative controls were performed by omitting the primary antibodies. Images were acquired with an AxioImager Z1 (Zeiss MicroImaging GmbH, Germany). All evaluations were performed in a blinded manner.

5 µm paraffin-sections of rat LV myocardium were stained with picrosirius red to obtain collagen content (52).

### RNA Extraction and Quantitative Real-Time PCR

The total RNA from cardiac rat tissue was isolated with RNA-Bee (Amsbio, UK) and a quantitative real-time PCR was performed with Brilliant SYBR Green qPCR master mix (Applied Biosystems, USA). The relative amount of target mRNA was determined using the comparative threshold (Ct) method as previously described (53). The mRNA contents of target genes were normalized to the expression of hypoxanthine phosphoribosyl transferase (HPRT).

### Protein Extraction and Immunoblotting

LV myocardium from male and female EAM rats was homogenized in Laemmli buffer (253mM Tris/HCL pH 6.8, 8% SDS, 40% glycerin, 200mM DTT, 0.4% bromophenol blue) (54). Proteins were quantified with the BCA Assay (Thermo Scientific Pierce Protein Biology, Germany). Equal amounts of total proteins were separated on SDS-polyacrylamide gels and transferred to a nitrocellulose membrane. The membranes were immunoblotted overnight with the following primary antibodies: Col3A1 (1:400, Santa Cruz, USA), ERK (1:1000, Santa Cruz, USA), p-ERK (1:2000, Santa Cruz, USA), p38 (1:500, Santa Cruz, USA) and p-p38 (1:500, Santa Cruz, USA). Equal sample loading was confirmed through an analysis of actin (1:1500, Santa Cruz, USA). Immunoreactive proteins were detected with ECL Plus (GE Healthcare, UK) and quantified with ImageLab (Bio-Rad Laboratories, USA).

### Statistical Analysis

All data are given as mean ± SEM. The data were evaluated with the non-parametric Mann-Whitney test for two independent groups or with two-way ANOVA analysis. Statistical analyses were performed with GraphPad Prism 5 (GraphPad Software, USA). Statistical significance was accepted when p < 0.05.

## Results

### Impaired Cardiac Function in Male EAM Rats

**Figure 1:**
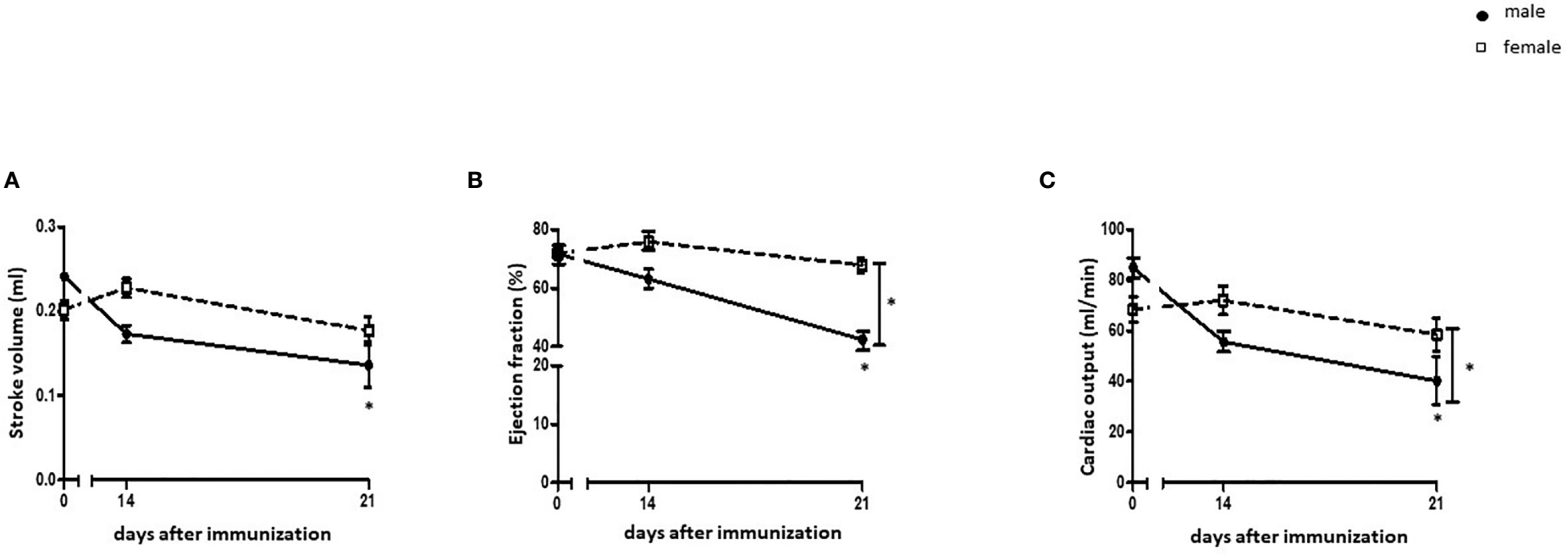
Cardiac function in EAM rats. Cardiac function parameters from the left ventricle were measured by CMR. **(A)** Stroke volume (SV) **(A)**, ejection fraction (EF) **(B)**, and cardiac output **(C)** were assessed before immunization, 14, and 21 days after immunization with cardiac myosin and CFA in male and female rats (n= 9-21). Data are shown as mean ± SEM. *p < 0.05.

Male rats showed a decline in stroke volume 21 days after immunization with cardiac myosin and CFA (p< 0.05), while no significant changes in female rats were detected (p> 0.05) (**Figure 1A**). Ejection fraction and cardiac output were significantly decreased in EAM male rats at 21 days after immunization in a sex-dependent manner (p< 0.05) (**Figures 1B, C**).

While male EAM rats had higher body and heart weights than female EAM rats (p< 0.05) (**Supplementary Figures 1A, B**), the relative heart weight to body weight ratio did not vary between sexes in the EAM rats (p> 0.05) (**Supplementary Figure 1C**).

The spleen, liver, and kidneys were significantly heavier in male EAM rats when compared to females, while the weight of the lungs was similar in both sexes (p< 0.05 and p> 0.05, respectively) (**Supplementary Figures 1D–G**).

### CD68+ ArgI+ Macrophages Are Increased in Myocardial Tissue in Female EAM Rats

**Figure 2:**
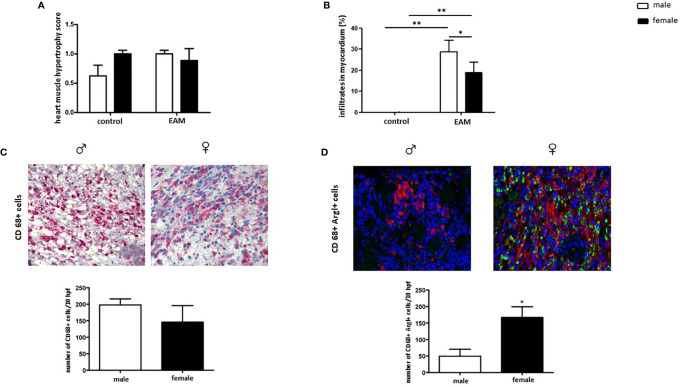
Increased number of cardiac CD68+ ArgI+ macrophages in female EAM rats. Immunohistochemical analysis of heart muscle hypertrophy **(A)**, myocardial immune infiltrates **(B)**, CD68+ immune-reactive cells **(C)**, and CD68+ ArgI+ cells **(D)** in myocardial tissue in male and female EAM animals. Data are shown as mean ± SEM (n= 4-12). *p < 0.05, **p < 0.01. Representative images of cardiac cryosections stained with antibodies against CD68 **(C)** and CD68 and ArgI **(D)** in myocardial tissue in male (♂) and female (♀) EAM animals (n= 4-12). Magnification 200x.

EAM rats did not show a higher immunohistochemical score for heart muscle hypertrophy when compared to healthy rats (p> 0.05) (**Figure 2A**). Despite an increased number of infiltrates detected both in male and female myocardial tissue after immunization (p< 0.01) (**Figure 2B**), female EAM rats showed significantly fewer immune cell infiltrates than male EAM rats (p< 0.05) (**Figure 2B**).

The number of cardiac CD68+ immune-reactive macrophages was similar in male and female EAM rats (p> 0.05) (**Figure 2C**). However, female EAM hearts had an increased number of cardiac anti-inflammatory CD68+ ArgI+ macrophages (p< 0.05) (**Figure 2D**), indicating an enhanced infiltration of M2 associated macrophages in females.

### Male EAM Rats Show More Fibrosis in Myocardial Tissue

To explore sex differences in collagen expression and fibrosis formation in EAM rats, the RNA and protein expression of collagen (Col3A1, Col1A1, Col4 and Col6), matrix metallopeptidase (MMP9), tissue metallopeptidase inhibitor 1 (TIMP1) and the pro-fibrotic factor, TGF-β were examined.

**Figure 3:**
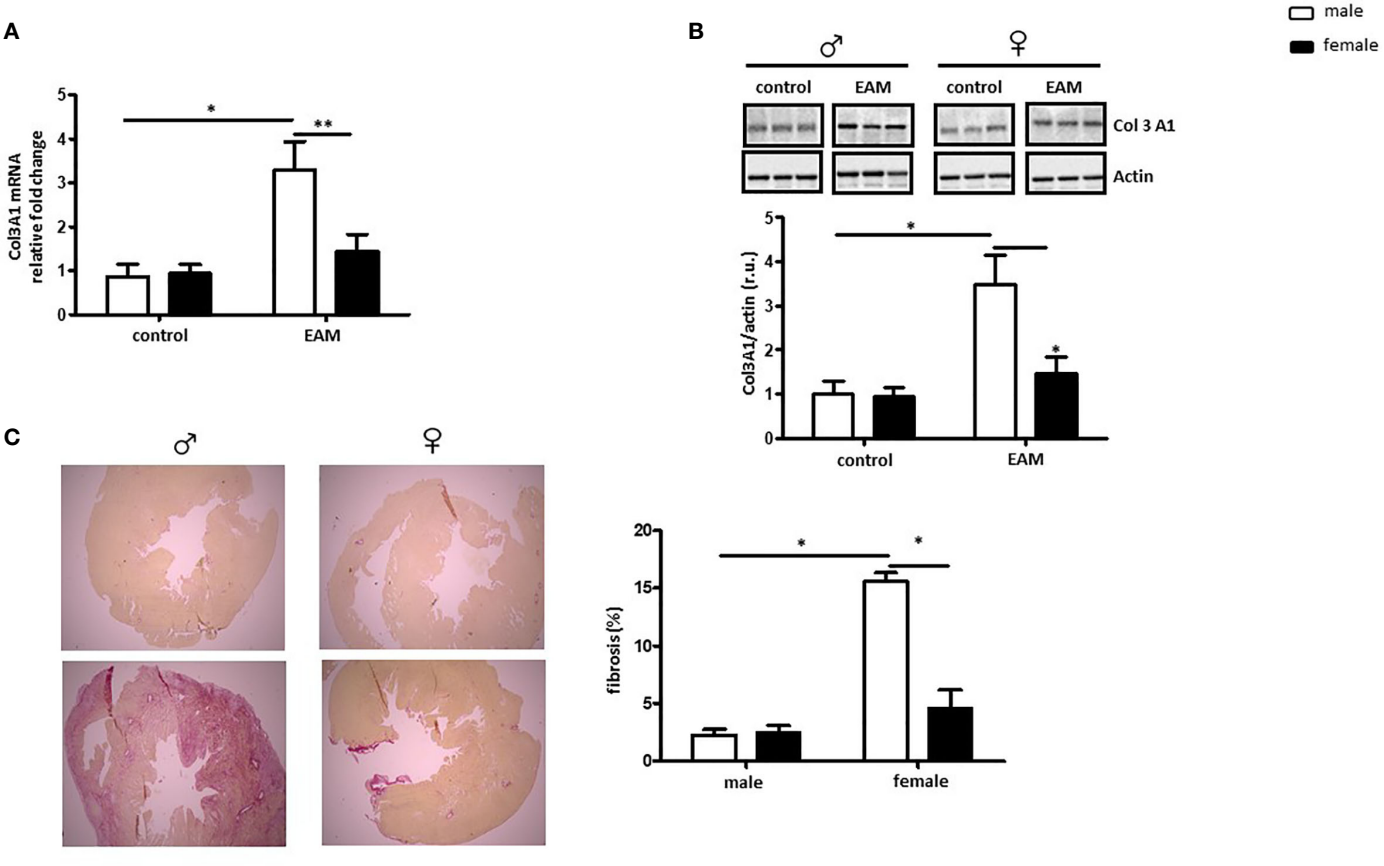
Male EAM rats develop more fibrosis in myocardial tissue. Analysis of Col3A1 mRNA **(A)** and protein expression **(B)** in cardiac tissue from control or EAM, male (♂) and female (♀). Data are shown as the mean ± SEM (n= 4-12). *p < 0.05, **p < 0.01. Representative imaging of western blot analysis; the lanes were run in the same gel. All data were normalized to the corresponding control and expressed in relative units (r.u.). **(C)** Representative Sirius red–dyed staining of cardiac tissue of 6 μm from male (♂) and female (♀) animals and corresponding statistics showing enhanced fibrosis in EAM rats (n= 4-12). Magnification 100x.

RNA and protein Col3A1 expression was significantly increased in the heart of EAM rats when compared to healthy controls in a sex-dependent manner (p< 0.05 and p> 0.05, respectively) (**Figures 3A, B**). Female EAM hearts had significantly less Col3A1 than males (p< 0.01 and p< 0.05) (**Figures 3A, B**). In accordance with these data, immunized male rats showed significantly higher amounts of fibrosis in comparison to female EAM hearts or non-immunized male hearts (p< 0.05) (**Figure 3C**).

Col1A1 mRNA expression was also significantly up-regulated in hearts from male but not female immunized rats (p< 0.05) (**Supplementary Figure 2A**). No changes in Col4 and Col6 expression were detected in EAM hearts (p> 0.05) (**Supplementary Figures 2B, C**). MMP-9 mRNA expression was up-regulated in female EAM hearts in comparison to male immunized rats (p< 0.05) (**Supplementary Figure 2D**). In addition, TIMP-1 expression was significantly up-regulated in female EAM hearts (p< 0.05 vs male EAM hearts) (**Supplementary Figure 2E**). Moreover, immunized female rats showed a significantly decreased TGF-β mRNA expression when compared with male immunized rats (p< 0.01) (**Supplementary Figure 2F**).

### Sex Differences in the Inflammatory Response in the EAM Model

Sex differences in the inflammatory response in myocarditis have been documented in both human and animal models (3). Both ERK and p38 activation are modulated *via* ER activation (55) and play a crucial role in the polarization of pro-inflammatory macrophages (56). Thus, we examined whether ERK and/or p38 activation (phosphorylation rate) are impaired in EAM hearts in a sex-dependent manner.

**Figure 4:**
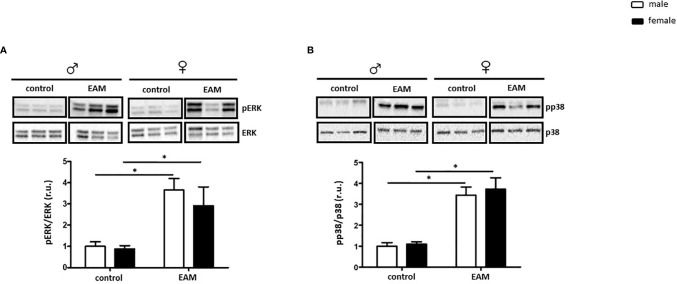
Sex-independent ERK and p38 activation in the EAM model. Western blot analysis of pERK/ERK ratio **(A)** and pp38/p38 ratio **(B)** in cardiac tissue lysates from control or EAM, male (♂) and female (♀). Data are shown as the mean ± SEM (n= 5-12). *p < 0.05. Representative imaging of western blot analysis; the lanes were run in the same gel. All data were normalized to the corresponding control and expressed in relative units (r.u.).

ERK phosphorylation was significantly increased in both male and female EAM rats (p< 0.05), while the amount of total ERK was unaffected (p> 0.05) (**Figure 4A**). In accordance with these findings, the pp38/p38 ratio was significantly increased in EAM rats in both sexes (p< 0.05) (**Figure 4B**). No significant changes in the p38 expression in EAM rats were found (p> 0.05) (**Figure 4B**).

**Figure 5:**
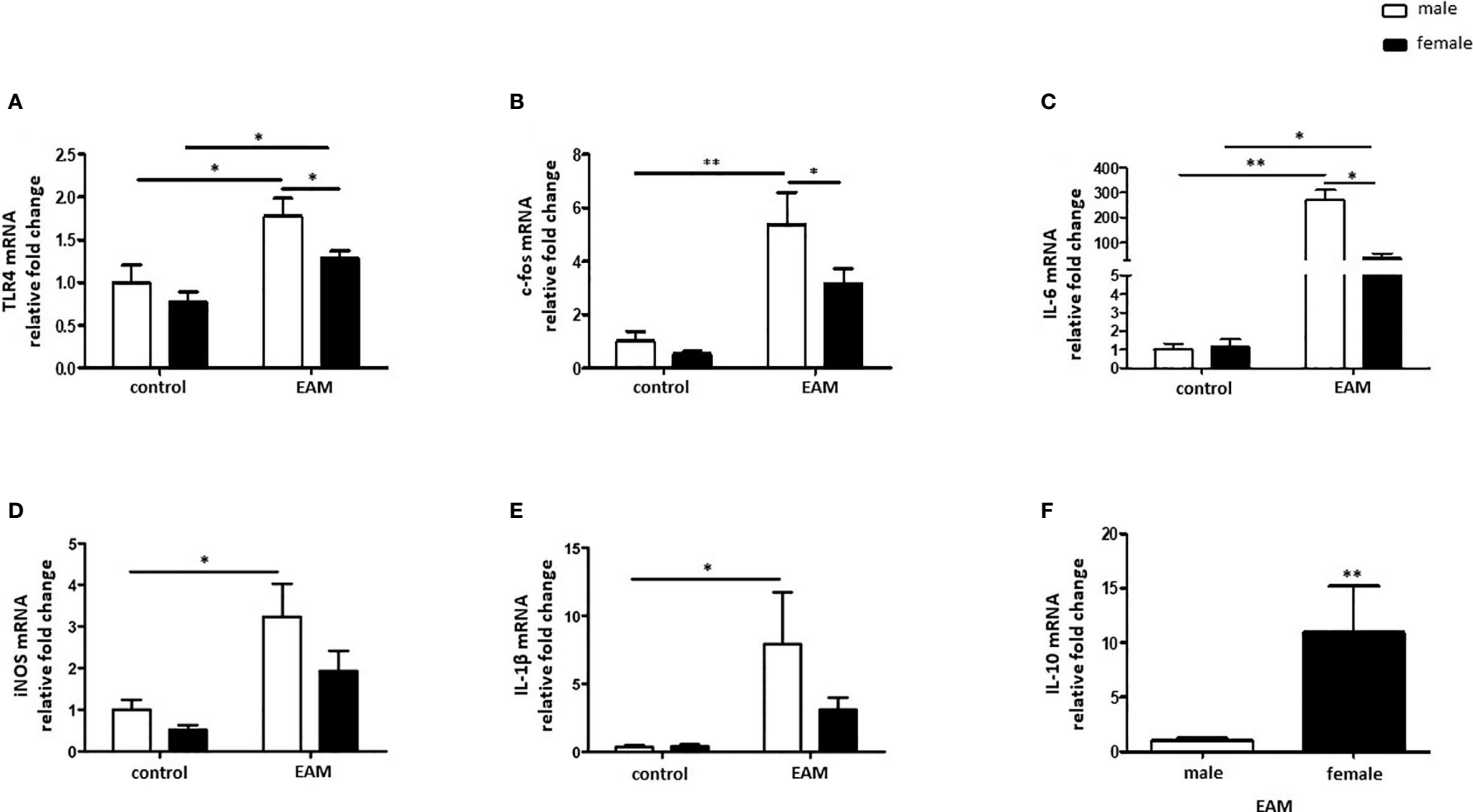
Sex differences in the inflammatory response in the EAM model. Real-time PCR analysis of TLR4 **(A)**, c-fos **(B)**, IL-6 **(C)**, iNOS **(D)**, IL-1β **(E)** and IL-10 **(F)** in rat cardiac tissue lysates from control or EAM, male (♂) and female (♀). Data are shown as the mean ± SEM (n= 5-12). *p < 0.05, **p < 0.01.

The mRNA expression of the pro-inflammatory marker TLR4 was significantly increased in both male and female hearts from EAM rats when compared to healthy hearts (p< 0.05) (**Figure 5A**), however TLR4 mRNA was significantly up-regulated in male EAM hearts in comparison to female EAM hearts (p< 0.05) (**Figure 5A**). Furthermore, the pro-inflammatory markers c-fos, IL-6, iNOS, and IL-1β were only up-regulated in hearts from male but not female EAM rats (p< 0.05) (**Figures 5B–E**). In accordance with these data, IL-10 mRNA expression was significantly up-regulated in immunized female rats in comparison to immunized male rats (p< 0.01) (**Figure 5F**). The expressions of TNF-α, NFκB, c-jun, and STAT1 were unchanged in both sexes after immunization (p> 0.05) (**Supplementary Figures 3A–D**).

## Discussion

In the current study, we investigated sex-dependent alterations in inflammation, collagen deposition and fibrosis formation in EAM rats. The main findings are: 1) Cardiac function was preserved in female rats after immunization, while the cardiac function was impaired in male EAM rats; 2) the number of cardiac anti-inflammatory CD68+ ArgI+ macrophages was only increased in female EAM rats; 3) collagen deposition and pathological fibrosis was only enhanced in hearts from male immunized rats; 4) pro-inflammatory mediators were significantly altered only in male EAM hearts. To summarize, an impaired inflammatory response and an exaggerated collagen deposition affecting the cardiac function were revealed in male EAM rats, while females demonstrated a protective response to adjuvant-induced EAM.

To the best of our knowledge, this is the first study to demonstrate sex differences in the inflammatory stage and in fibrosis formation, with a decline in cardiac function in an EAM rat model.

In clinical setting, men are more likely to develop myocarditis and DCM than women (17, 18, 57–59). More pronounced inflammation and fibrosis have been reported in male individuals with myocarditis than in female individuals (21, 22). A potential contribution of sex hormones may be a factor, as the association of DCM and heart failure with high testosterone levels has been previously reported (25, 60–62). These sex differences in humans correspond to sex differences in the mouse model. It is also interesting to note that female mice develop less inflammation after infection with CVB3 by similar viral replication (23, 24).

The EAM immunization protocol is used as a model of the chronic inflammatory phase of post-viral myocarditis (13), characterized by ongoing inflammation, fibrotic remodeling, appearance of anti-myosin antibodies, and development of DCM in the end-stage (63). Schmerler et al. have shown that male EAM rats had decreased ejection fraction and stroke volume (13). In keeping with those results, in our study the ejection fraction and the stroke volume showed a prominent decline in male EAM animals but no significant changes in females were detected, suggesting a preserved cardiac function in females.

Male EAM rats developed autoimmune myocarditis 21 days after immunization with cardiac myosin and CFA in the paw, accompanied by an increased amount of myocardial immune cell infiltrates and CD68+ immune reactive cells (13). In accordance with this study, we detected an increased number of immune cell infiltrates in the heart of male rats after immunization. In female EAM rats, although the infiltrates in the myocardial tissue were increased, it was significantly less than in male rats, indicating a weaker immune response in females.

Macrophages are the central regulator of the immune system in the heart in a normal state as well as during cardiac inflammation (64), and their crucial role in pro-fibrotic processes during chronic inflammation has been reported elsewhere (65). Though we found no sex differences in the number of immune reactive CD68+ macrophages in our EAM model, hearts from female EAM rats were infiltrated with an increased amount of anti-inflammatory CD68+ ArgI+ macrophages, suggesting that a predominant phenotype in females is alternative activated macrophages (M2) that favor an anti-inflammatory environment thus attenuating inflammation in female hearts in autoimmune myocarditis. However, M2 macrophages seem to be involved in the production of collagen and fibrosis formation (66), associated with an increased arginase activity (67). In agreement with this, sex differences in viral myocarditis and post-myocarditis complications, e.g., development of cardiac autoimmunity and DCM, are not caused by the virus itself, but rather by sex-related differences in the immune response (11). Moreover, Fairweather et al. have shown that, in a viral induced myocarditis model, the detrimental immune response in male individuals is driven by a predominant M1 response, while female animals show a stronger M2 response (38, 68). Our results suggest that macrophage polarization plays a crucial role in the development of sex differences in cardiac inflammation. The activation of the M2 response counteracts the detrimental effects of the pro-inflammatory macrophage polarization during acute inflammation, suggesting that a predominant M2 response is cardio protective (42). Our results indicate a pro-inflammatory M1-mediated and M2-mediated anti-inflammatory immune reaction in the heart of male and female rats, respectively.

Chronic activation of the inflammatory response leads to increased collagen deposition and pathological fibrosis is part of many diseases including myocarditis (69). Here it is important to remember that macrophages play a key role in the regulation of fibrosis (70) and activate fibroblasts *via* TGF-β, platelet-derived growth factor (PDGF) and TNF-α (47, 48). In our study, we detected an increased expression of Col3A1 and Col1A1 in the cardiac tissue from male EAM rats, while female EAM rats expressed similar amounts of Col3A1 and Col1A1 as healthy rats. In accordance, the anti-fibrotic factor, TIMP1 was up-regulated in immunized female rats, while the pro-fibrotic factor, TGF-β was decreased in females. Additionally, male EAM rats develop pathological fibrosis in the heart after immunization, while female EAM rats do not, suggesting that they undergo a different, fibrosis-independent, immune response. Indeed, severe fibrosis was previously reported in hearts from males with EAM (13), which may potentially be caused by increased testosterone levels (25–28, 71).

Enhanced ERK and p38 activity was detected in EAM rats in comparison to non-immunized rats of both sexes, arguing that other cascades are involved in activation of pro-inflammatory mediators in male EAM rats. In fact, a stronger M1 response and altered pro-inflammatory mediators were demonstrated solely in male animals. Male EAM rats showed an increased expression of TLR4, IL-6, c-fos, and iNOS when compared to healthy animals, while no significant changes were detected in female EAM rats, indicating that females did not develop a pro-inflammatory response after immunization. In accordance, Roberts et al. demonstrated sex-differences in the cardiac TLR4 expression in CVB3 infected mice, increasing the pathogenicity in male but not female infected mice (72). Of note, c-fos is a key transcription factor for the M1 spectrum, and iNOS is a signature M1 enzyme, reinforcing the observation of sex-dependent macrophages polarization in EAM (42, 73, 74).

Fairweather et al. have proposed a pivotal role for sex hormones in the sex-related differences in cardiac inflammation (75). While estrogen has cardio-protective properties in females, characterized by reducing cardiomyocyte apoptosis, counteracting fibrosis (76, 77), and deactivating cellular pathways that induce hypertrophy (17, 78, 79), testosterone increased cardiac inflammation in a myocarditis mice model (24) and encouraged a M1 response of macrophages in male individuals (25). Moreover, Koenig et al. also reported pro-inflammatory actions of androgens and anti-inflammatory actions of estrogen in CVB3 induced experimental myocarditis (80). Recent studies have also demonstrated that estrogen directly regulates macrophage polarization in different pathological tissue states (81–83), suggesting that E2 is directly involved in the polarization into M2 macrophages in female EAM rats. However, the spectrum of macrophage phenotypes to be researched is larger (42) and the role of sexual hormones should be investigated in the EAM model more in depth.

In conclusion, the present study revealed that autoimmune myocarditis is associated with an increased pro-inflammatory response in males, leading to fibrotic formation, while in females the model is associated with a muted pro-inflammatory response, balanced immune-regulation, and preserved cardiac function.

## Data Availability Statement

The original contributions presented in the study are included in the article/**Supplementary Material**. Further inquiries can be directed to the corresponding author.

## Ethics Statement

The animal study was reviewed and approved by Landesamt für Gesundheit und Soziales, Berlin.

## Author Contributions

MB conceived the project, analyzed the data, prepared the figures, and wrote the main manuscript text. MN performed the molecular experiments and analyzed the data. SJ generated the model, performed the functional experiments, obtained the tissue, and analyzed data. SP analyzed the data and wrote the main manuscript text. NH performed molecular experiments and analyzed the data. AK performed the immunohistochemical experiments, analyzed the data, and revised the manuscript. DM designed the functional experiments, provided the EAM tissue, and revised the manuscript. VR-Z generated research funds, initiated, and coordinated the project. SJ and VR-Z revised the manuscript. All authors contributed to the article and approved the submitted version.

## Funding

Parts of this work were funded by the DZHK (German Centre for Cardiovascular Research) and by the BMBF (German Ministry of Education and Research). We acknowledge support from the German Research Foundation (DFG) and the Open Access Publication Fund of Charité – Universitätsmedizin Berlin.

## Conflict of Interest

The authors declare that the research was conducted in the absence of any commercial or financial relationships that could be construed as a potential conflict of interest.

## Supplementary Material

Supplementary Figure 1 Sex differences in heart weight to body weight ratio. Body weight (BW) **(A)** and heart weight (HW) **(B)** and relative heart weight to body weight (HW/BW) **(C)** were measured 21 days after immunization with cardiac myosin and CFA. In addition, the weight from spleen **(D)**, lung **(E)**, liver **(F)**, and both kidneys **(G)** was assessed 21 days after immunization with cardiac myosin and CFA (n= 4-12). Data are shown as mean ± SEM. *p< 0.05, **p< 0.01.

Supplementary Figure 2 Sex differences in the expression of pro- and anti-fibrotic factors in EAM. Real-time PCR analysis for Col1A1 **(A)**, Col4 **(B)**, Col6 **(C)**, MMP9 **(D)**, TIMP1 **(E)** and TGF-β **(F)** performed with rat cardiac tissue from control or EAM male (♂) and female (♀). Data are shown as the mean ± SEM (n= 4-12). *p< 0.05, **p< 0.01.

Supplementary Figure 3 TNF-α and NFκB are not increased in EAM. Real-time PCR analysis for TNF-α **(A)**, NFκB **(B)**, c-jun **(C)** and STAT1 **(D)** performed with rat cardiac tissue from control or EAM, male (♂) and female (♀). Data are shown as the mean ± SEM (n= 5-12).
