# Supplementary figures and images for: Sex-Specific Differences of the Inflammatory State in Experimental Autoimmune Myocarditis
Source: Front Immunol. 2021 May 28;12:686384. doi: 10.3389/fimmu.2021.686384 (PMC8195335; doi:10.3389/fimmu.2021.686384)

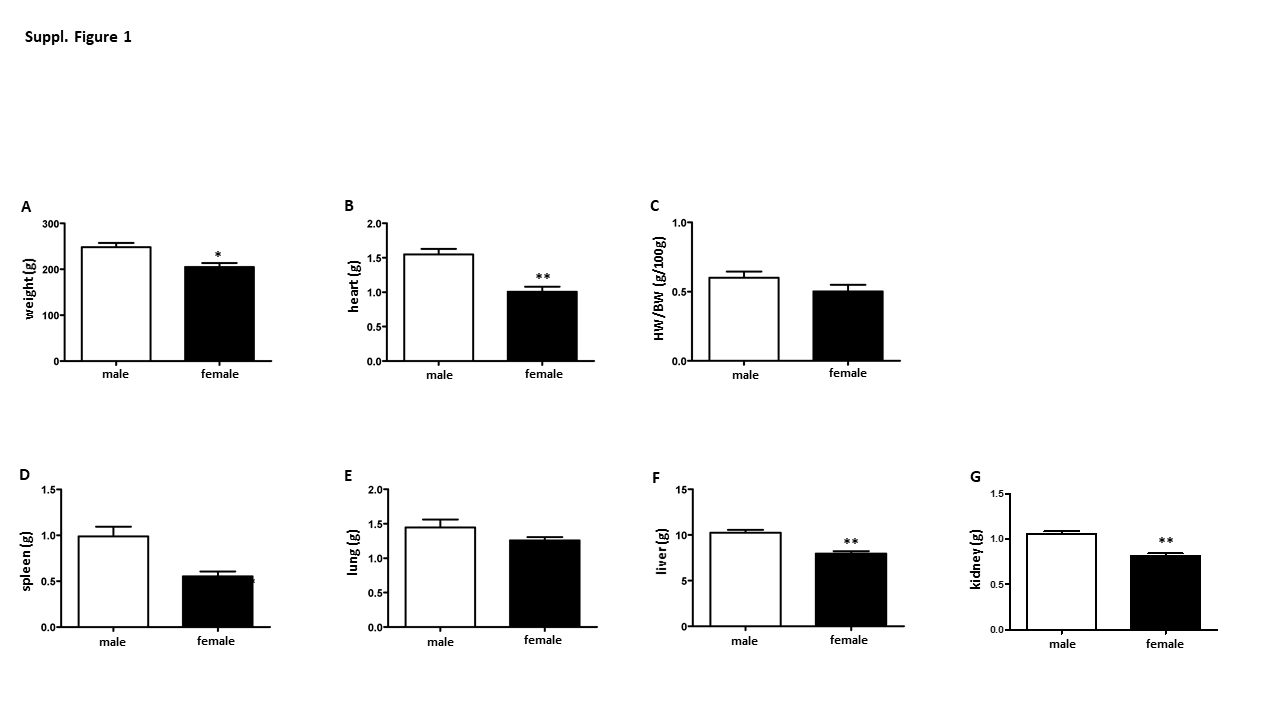

Supplement: Supplementary Figure 1 — Sex differences in heart weight to body weight ratio. Body weight (BW) (A) and heart weight (HW) (B) and relative heart weight to body weight (HW/BW) (C) were measured 21 days after immunization with cardiac myosin and CFA. In addition, the weight from spleen (D), lung (E), liver (F), and both kidneys (G) was assessed 21 days after immunization with cardiac myosin and CFA (n= 4-12). Data are shown as mean ± SEM. *p< 0.05, **p< 0.01. [file Image_1.tif]

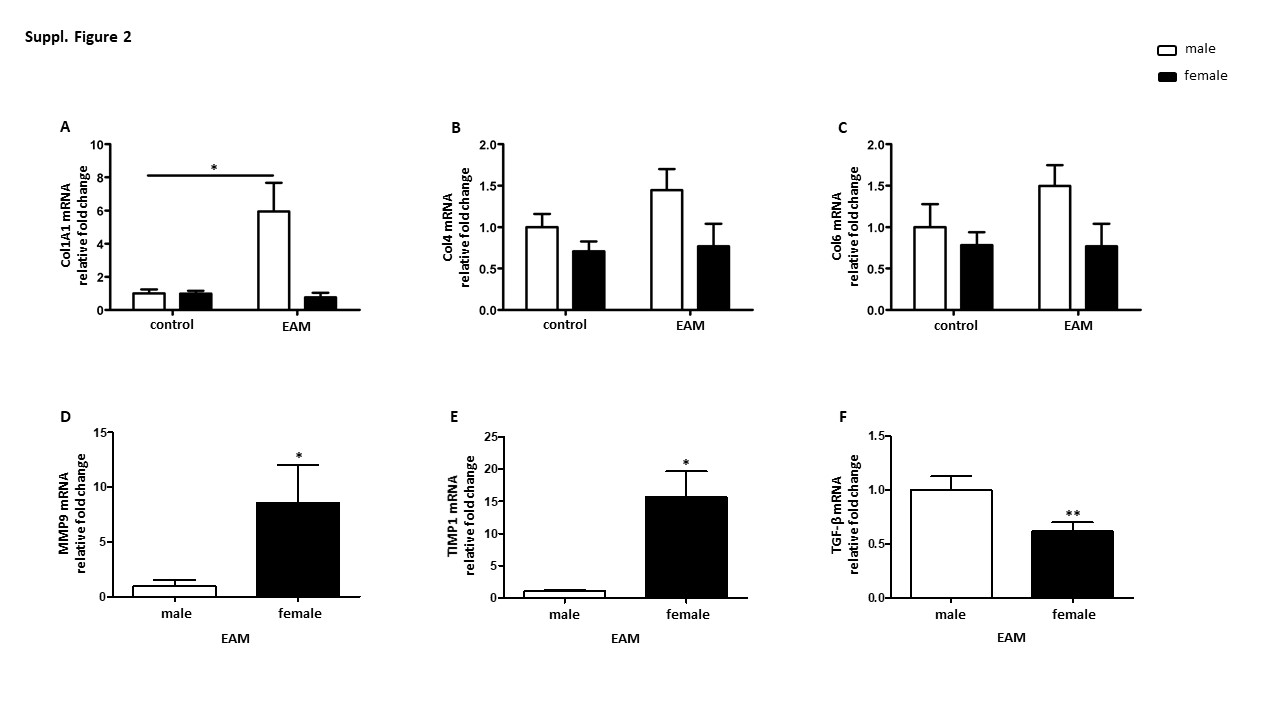

Supplement: Supplementary Figure 2 — Sex differences in the expression of pro- and anti-fibrotic factors in EAM. Real-time PCR analysis for Col1A1 (A), Col4 (B), Col6 (C), MMP9 (D), TIMP1 (E) and TGF-β (F) performed with rat cardiac tissue from control or EAM male (♂) and female (♀). Data are shown as the mean ± SEM (n= 4-12). *p< 0.05, **p< 0.01. [file Image_2.jpg]

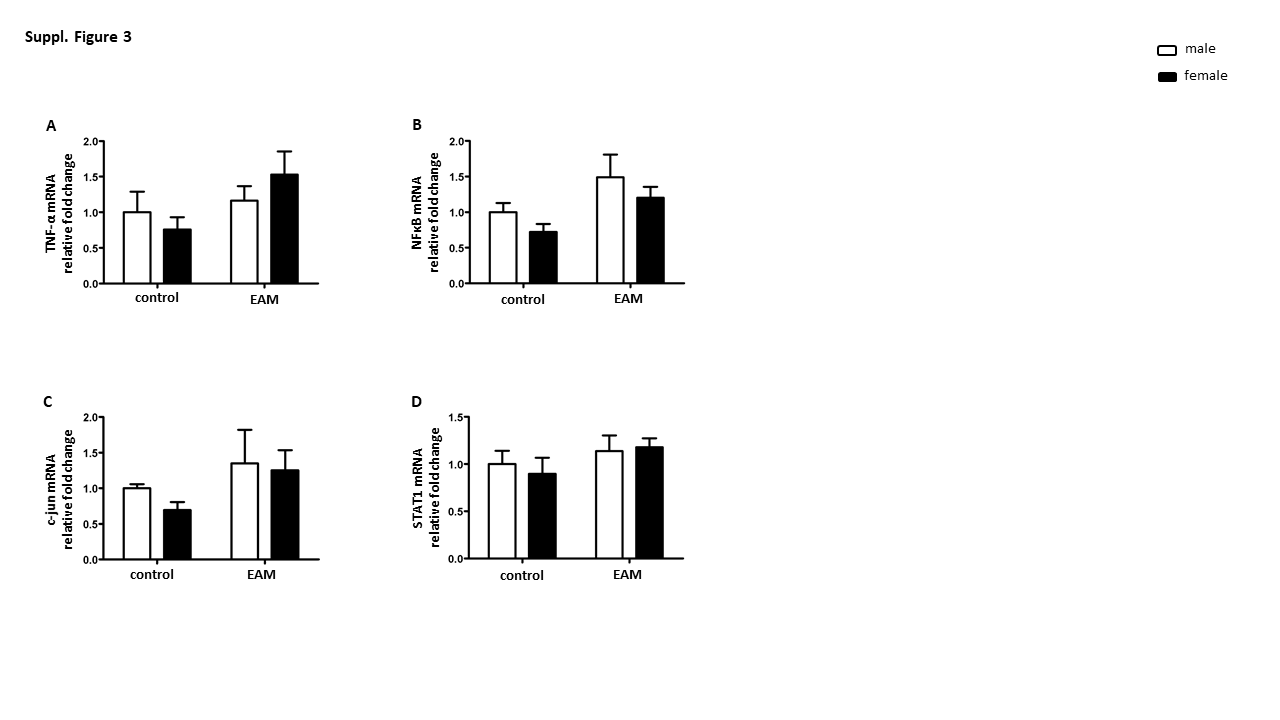

Supplement: Supplementary Figure 3 — TNF-α and NFκB are not increased in EAM. Real-time PCR analysis for TNF-α (A), NFκB (B), c-jun (C) and STAT1 (D) performed with rat cardiac tissue from control or EAM, male (♂) and female (♀). Data are shown as the mean ± SEM (n= 5-12). [file Image_3.tif]
